# Research and Implementation of Millet Ear Detection Method Based on Lightweight YOLOv5

**DOI:** 10.3390/s23229189

**Published:** 2023-11-15

**Authors:** Shujin Qiu, Yun Li, Jian Gao, Xiaobin Li, Xiangyang Yuan, Zhenyu Liu, Qingliang Cui, Cuiqing Wu

**Affiliations:** 1 College of Agricultural Engineering, Shanxi Agriculture University, Jinzhong 030801, China; s20202059@stu.sxau.edu.cn (Y.L.); z20223672@stu.sxau.edu.cn (J.G.); lixiaobin@sxau.edu.cn (X.L.); lzysyb@sxau.edu.cn (Z.L.); cuiqingliang@sxau.edu.cn (Q.C.); cqwu@sxau.edu.cn (C.W.); 2 College of Agricultural, Shanxi Agricultural University, Jinzhong 030801, China; yuanxiangyang200@sxau.edu.cn

**Keywords:** millet ear, YOLOv5, lightweight model, algorithmic optimization, Jetson Nano

## Abstract

As the millet ears are dense, small in size, and serious occlusion in the complex grain field scene, the target detection model suitable for this environment requires high computing power, and it is difficult to deploy the real-time detection of millet ears on mobile devices. A lightweight real-time detection method for millet ears is based on YOLOv5. First, the YOLOv5s model is improved by replacing the YOLOv5s backbone feature extraction network with the MobilenetV3 lightweight model to reduce model size. Then, using the multi-feature fusion detection structure, the micro-scale detection layer is augmented to reduce high-level feature maps and low-level feature maps. The Merge-NMS technique is used in post-processing for target information loss to reduce the influence of boundary blur on the detection effect and increase the detection accuracy of small and obstructed targets. Finally, the models reconstructed by different improved methods are trained and tested on the self-built millet ear data set. The AP value of the improved model in this study reaches 97.78%, F_1_-score is 94.20%, and the model size is only 7.56 MB, which is 53.28% of the standard YoloV5s model size, and has a better detection speed. Compared with other classical target detection models, it shows strong robustness and generalization ability. The lightweight model performs better in the detection of pictures and videos in the Jetson Nano. The results show that the improved lightweight YOLOv5 millet detection model in this study can overcome the influence of complex environments, and significantly improve the detection effect of millet under dense distribution and occlusion conditions. The millet detection model is deployed on the Jetson Nano, and the millet detection system is implemented based on the PyQt5 framework. The detection accuracy and detection speed of the millet detection system can meet the actual needs of intelligent agricultural machinery equipment and has a good application prospect.

## 1. Introduction

Millet is one of the most important miscellaneous grain crops in China. Its planting area accounts for around 80% of the world’s total planting area, while its output accounts for approximately 90% of the world’s total output [1]. For a long time, the number of ears had to rely on manual observation and statistics in the study of millet cultivation and breeding, which is labor-intensive, time-consuming, and inefficient. In the actual mixed environment, the similarity, dense distribution, occlusion, and subjectivity of statisticians make counting grains and ears difficult, and mistakes are common. Millet ears are a key agronomic index to evaluate the yield and quality of foxtail millet, which plays an important role in nutritional diagnosis, growth period detection, and pest detection. Therefore, rapid and accurate detection of millets on mobile devices can play an important role in yield estimation and phenotypic research. With the rapid development of agricultural information technology, crop image detection based on deep learning has received extensive attention [2,3].

At present, the research on grain spike detection is mainly based on wheat [4,5,6], rice [7,8,9,10], and other major grain crops. The research problems are mainly aimed at improving the detection accuracy and detection speed of the model. Bao et al. [11] proposed a wheat spike recognition model based on convolutional neural network. In order to improve the recognition accuracy, a sliding window was constructed by image pyramid to realize multi-scale recognition of wheat spikes. The accuracy of the model was 97.30%. The model was used to complete the counting of wheat spikes and estimate wheat yield. Zhang et al. [12] realized a convolutional neural network recognition model for winter wheat spikes and combined it with non-maximum suppression values to achieve rapid and accurate detection of wheat spikes in the actual environment. Wang et al. [13] realized the detection and counting of wheat spike targets in different periods by improving the YOLOv3 model. The detection results of the improved YOLOv3 model showed strong robustness, but it was still difficult to detect occluded wheat spikes and smaller wheat spikes. The research of Bao et al. [14] based on the deep convolutional neural network CSRNet network studied the density map of a single wheat ear and counted the wheat ears according to the density value. Xu et al. [15] adopted the minimum area intersection ratio (MAIR) feature extraction algorithm and the transfer learning technology to achieve automatic wheat ear counting based on the YOLOv5 model. Liu et al. [16] used the improved Bayes matting algorithm to segment the wheat ear from the complex background, and used smoothing filtering, erosion, filling, and other algorithms to segment the wheat ear spikelets and form a connected area for marking and counting. This method improves the technical accuracy. Xie et al. [17] proposed a wheat ear detection model based on deep learning (FCS R-CNN) and introduced methods such as feature pyramid network (FPN) through Cascade R CNN to improve detection accuracy and detection speed.

In the actual environment, the millet ear is densely distributed and seriously occluded, and the model is difficult to detect the ear head in a complex environment. Therefore, when designing the model, it is necessary to consider the model’s blurring of small-scale targets, occlusion targets, and target boundaries, as well as the deployment of the model on the embedded platform to the actual environment. Jiang et al. [18] designed a rice panicle detection method based on generating feature pyramid (GFP-PD). Aiming at the noise of small-sized rice panicles and leaves blocking rice panicles, the structural feature pyramid and occlusion sample repair module (OSIM) were used to improve the detection accuracy of the model. Zhang et al. [19] introduced dilated convolution based on the Faster R-CNN model to solve the problem of small-sized rice panicle target and used ROIAign instead of ROIPooling to optimize and improve the average detection accuracy of the model for rice panicles. Jiang et al. [20] proposed an improved NMS-based max intersection over portion (MIoP-NMS) algorithm and implemented it in the YOLOv4 network framework for single-stage target detection, and estimated the number of banana trees in dense occluded banana forests with about 98.7% accuracy. Bao et al. [21] designed a lightweight convolutional neural network simple net, which is constructed using convolution and reverse residual blocks, and combined it with the convolutional attention mechanism CBAM module, which can be used for automatic recognition of wheat ear diseases on mobile terminals. Zhao et al. [22] proposed an improved YOLOv5-based method to detect wheat ears in UAV images. By adding a micro-scale detection layer and using the WBF algorithm, the detection problem caused by the dense distribution and occlusion of small-sized wheat ears was solved. Yang et al. [23] proposed an improved YOLOv5 apple flower growth state detection method, introduced CA attention module, and designed multi-scale detection structure to improve the detection accuracy of the model. Zhang et al. [24] designed a potato detection model by improving the YOlOv4 model. The CSP-Darknet53 network of the YOLOv4 model was replaced by the MobilenetV3 network to reduce the model volume and ensure the average detection accuracy of the potato. The experiment was deployed on embedded devices, and YOLOv4-MobilenetV3 showed strong robustness.

Due to the growth characteristics of millet in the natural environment, the shape and spatial distribution of millet ears are irregular, so it is difficult to apply the target detection model to detect the millet ears in the actual environment. In this study, the YoloV5s model was used as the original model, and the main feature extraction network is replaced by the lightweight MoblienetV3 model to reduce the model size. On this basis, the feature fusion detection structure is improved, and the Merge-NMS algorithm is used to improve the lightweight model. By testing and evaluating the model on the self-built grain data set, it provides a theoretical basis for rapid and accurate detection of grain on mobile devices.

## 2. Materials and Methods

### 2.1. Image Acquisition

**Figure 1:**
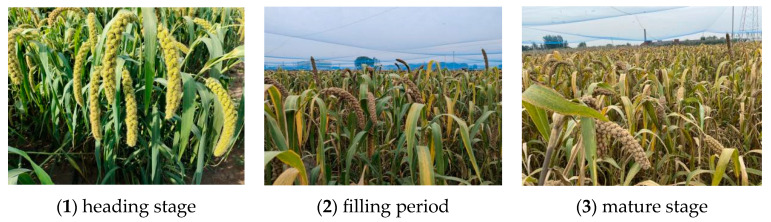
Data set of millet ear in different stages.

The millet ear images were collected from the experimental field of Shenfeng Village, Shanxi Agricultural University. The millet ear images (Figure 1) included 25 heading stage, 230 filling stage, and 45 mature stage, for a total of 300 images. The length of millet ear is about 20–35 cm, the growth state is inclined to one side along the end of the stem, the millet ear head is downward, and the planting density of millet is very high, about 375,000 to 600,000 plants/ha, resulting in serious occlusion of the millet ears in the field, which affects the detection effect of the traditional target detection model on the millet ears. Therefore, the images were taken from the upper side in this study. The resolution of the collected grain image is 4032 pixels × 3024 pixels, which is stored in the jpg format. Due to the limited computing resources in the laboratory, the original image is compressed to 1024 pixels × 768 pixels to speed up the data processing time. There are many complex situations in the millet images collected in the natural environment, such as grains covered by leaves and stems, grains intertwined with each other, dense distribution of grains, etc., which have certain interference in the detection of grains by the model.

### 2.2. Image Preprocessing

**Figure 2:**
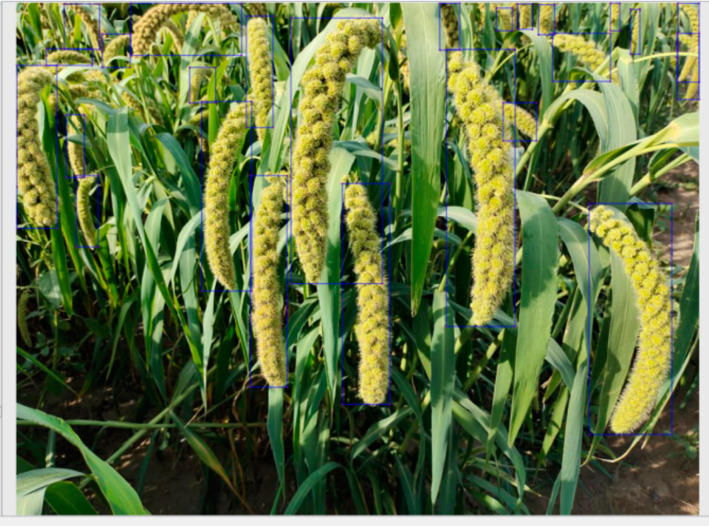
Millet ears annotation.

**Figure 3:**
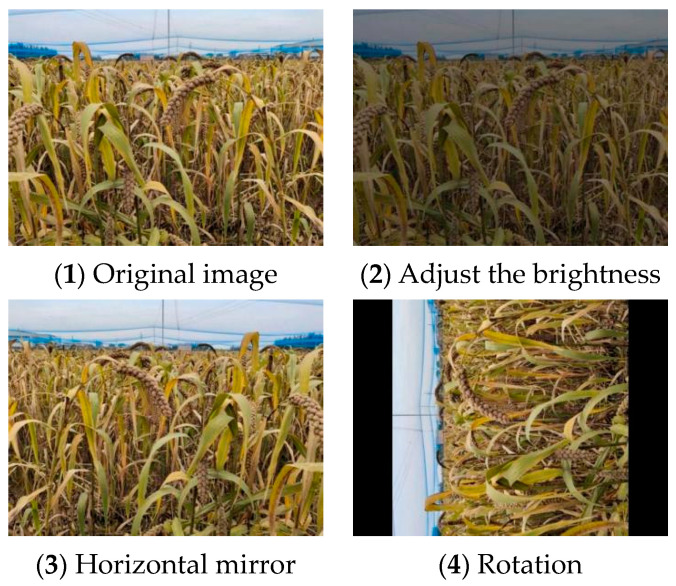
Millet ear image data set enhancement.

LabelImg annotation tool is used to make the grain image data set according to the PASCAL VOC data set format for the collected millet image, and the grain in the image is marked (Figure 2) to generate the corresponding XML file. In order to prevent the over-fitting of the network model caused by the small data set and improve the generalization ability of the network model training results, it is necessary to use data augmentation for the millet ear data set (Figure 3). In this study, the self-made grain and millet data set was randomly enhanced by rotation, flipping, mirroring, brightness adjustment, and other methods. The annotation files corresponding to each image were transformed at the same time, and the data set was expanded to 2100. The data set was randomly divided into a training set, verification set, and test set according to the ratio of 8:1:1.

### 2.3. YoloV5 Model and Its Improvement

#### 2.3.1. YoloV5 Model

**Figure 4:**
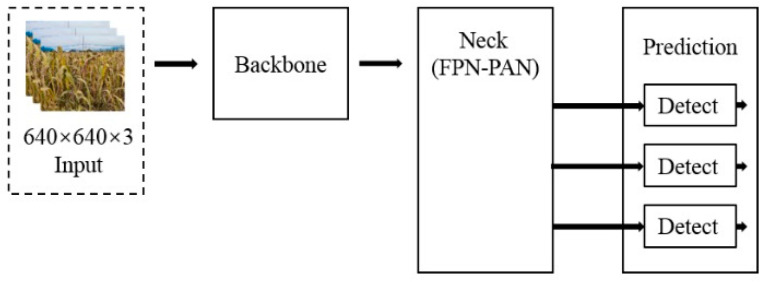
The structure of YoloV5s model.

The YOLO (You Only Look Once) [25] series is a single-stage target detection model using regression method with good performance. As shown in Figure 4, the structure of the YoloV5 model is shown. The YoloV5s model mainly includes input, backbone, neck, and prediction. The backbone structure is used as a feature extraction and convolution operation of different times to determine the model complexity and parameter quantity.

**Figure 5:**
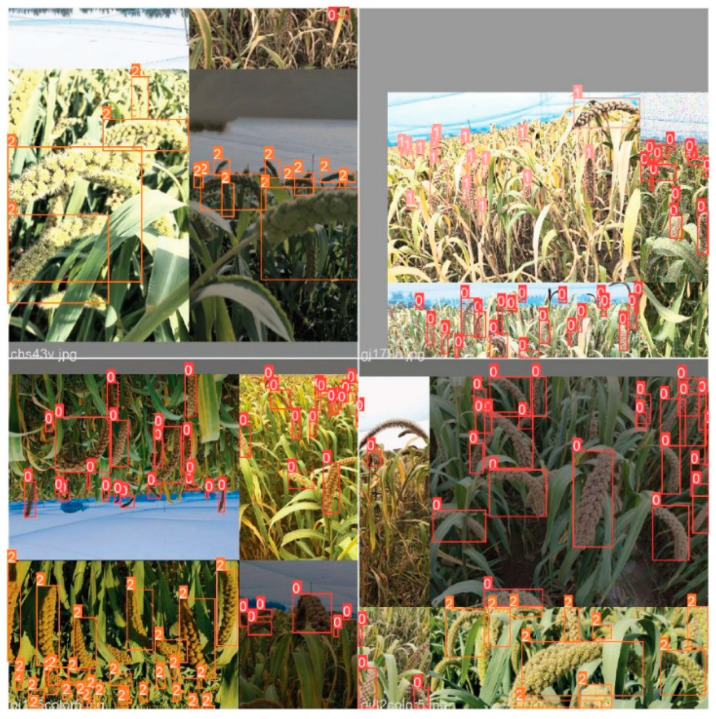
Mosaic data augmentation. Note: 0 is millet ears in the heading stage, 1 is the mature stage, and 2 is the filling stage.

The YoloV5s input retains the same mosaic data augmentation method as YOLOv4, and randomly scales, cuts, distributes, and splices the four images into a new picture, as shown in Figure 5.

The backbone of YoloV5s adds the Focus structure to realize the slicing operation of the input image. The size of the input feature map is 640 × 640 × 3. The size of the output feature map obtained by the Focus structure is 320 × 320 × 32. The backbone network follows the cross-level partial network (CSP) structure of YOLOv4, and mainly uses the residual network structure to extract the features of the input image, in which the convolution operation determines the complexity and parameter quantity of the whole model [26].

The neck uses FPN-PAN structure. The feature pyramid network FPN (FPN) transmits and fuses the high-level feature information and the information of the backbone feature extraction network from top to bottom through up sampling. The pyramid attention network (PAN) structure transmits the target positioning feature from bottom to top through down sampling. The combination of the two improves the detection ability of the model. The bounding box loss function of Prediction uses the CIOU_LOSS (Complete IoU Loss) function and the non-maximum suppression (NMS) method to effectively obtain the best prediction anchor box.

YOLOv5 uses the gradient descent method to optimize the objective function during the training process. As the number of iterations increases, the loss value (LOSS) is close to the global minimum, and the learning rate is also small. In order to make the model reach the best convergence state after training, the cosine annealing learning rate adopted by YOLOv5 is to reduce the learning rate through the cosine function. The cosine function value decreases slowly with the increase of x, then rises rapidly, and then decreases slowly. The purpose is to avoid falling into the current local optimal point, and constantly adjust the learning rate to make the model converge to a new optimal point until the model training stops. The principle of cosine annealing learning rate is as follows:

(1) $$l_{new}=l_{\min}^{i}+\frac{1}{2}\left(l_{\max}^{i}-l_{\min}^{i}\right)\left(1+\cos\left(\frac{T_{cur}}{T_{i}}\right)\right)$$

where *l*_new_ is the latest learning rate, *i* is the number of executions (index value), $l_{\min}^{i}$ is the minimum learning rate, $l_{\max}^{i}$ is the maximum learning rate, *T*_cur_ is the number of epochs currently executed, and *T*_i_ is the total number of epochs in the execution *i*.

#### 2.3.2. Improvement of YoloV5 Model

##### Use MobilenetV3 to Modify the Model Structure of YoloV5

MoblienetV3 [27] is a lightweight neural network that combines real time, speed, and accuracy. The backbone network of MoblienetV3 is based on the Bneck structure composed of inverted residual blocks, including ordinary convolution, and deep separable convolution, and adds an attention mechanism (SE module) to the fully connected layer. Compared with the standard convolution, the depthwise separable convolution in the inverted residual block can significantly reduce the number of parameters of the overall model and reduce the model size [28].

**Figure 6:**
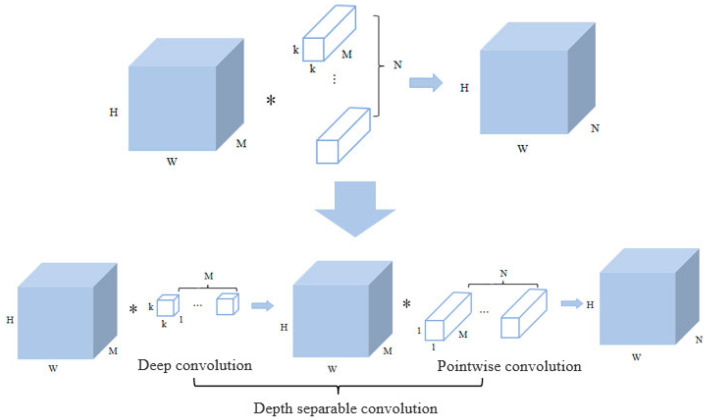
The depth separable convolution. Note: The ‘*’ representing the multiplication of two convolutions.

As shown in Figure 6, assuming that the size of the input feature map is *H* × *W* × *M* (channel is *M*), the size of the output feature map is *H* × *W* × *N* (channel is *N*) after *N* standard convolutions of *k* × *k* × *M*.

The parameters of standard convolution are calculated as follows:

(2) $$P_{1}=k\times k\times M\times N=k^{2}\times M\times N$$

The depth separable convolution is composed of deep convolution and pointwise convolution. The convolution kernel size of the deep convolution is *k* × *k* × 1, and there are *M* convolution kernels, which are responsible for filtering each channel of the input. The convolution kernel of point-by-point convolution is 1 × 1 × *M*, which has *N* convolution kernels and is responsible for converting channels. The parameters of depth separable convolution are calculated as follows:

(3) $$P_{2}=k\times k\times 1\times M+1\times 1\times M\times N=k^{2}\times M+M\times N=M\times (k^{2}+N)$$

Therefore, the depth separable convolution is compared with the standard convolution parameter as follows:

(4) $$\frac{P_{2}}{P_{1}}=\frac{M\times \left(k^{2}+N\right)}{k^{2}\times M\times N}=\frac{1}{N}+\frac{1}{k^{2}}$$

##### Merge-NMS Algorithm

The influence of the resolution of the image will reduce the detection performance, i.e., the blurred pixels of the image will lead to the problem of blurred boundary of the detection target. Due to this factor, it is not easy to accurately distinguish overlapping and occluded millets. In this study, the standard non-maximum suppression value (NMS) was improved to the fusion non-maximum suppression value (Merge-NMS) [29] to reduce the blurred grain target boundary in the post-processing process. At the end of each iteration, the standard NMS only retains the anchor box with the highest score, and the anchor boxes that overlap with this anchor box will be suppressed, and a large number of valuable anchor boxes will also be suppressed. Merge-NMS utilizes the anchor frame information suppressed by the standard NMS and fuses it with other anchor frames to obtain a more accurate prediction anchor frame. The box in the pseudo-code of Merge-NMS is the detection anchor box, *Cls* is the classification confidence, and *Loc* is the location confidence. The final score *S* of the anchor box is obtained by multiplying *Cls* and *Loc*. At the beginning, all anchor boxes are sorted according to the score *S*. In each cycle, the anchor frame (*b*_m_) with the highest score is taken out from all anchor frames. If the score of the anchor frame highly overlapped with bm is greater than the threshold of Merge-NMS, bm will merge with these frames to form a new detection anchor frame and put it into the final detection set D. The new detection anchor frame calculation method is as follows:

(5) $$x_{m}=\frac{\sum_{k}loc_{k}\times x_{k}}{\sum_{k}loc_{k}}$$

where *x_m_* is the coordinate of *b*_m_, *loc_k_* is the location confidence of k, and *x_k_* is the coordinate of the selected anchor frame in each cycle.

The higher the location confidence is, the higher the weight of the anchor frame of *loc*_k_ in the new detection anchor frame *x_m_*.

##### Improvement of Multi-Feature Fusion Detection Structure

The original structure of YoloV5s is designed with three scale feature detection layers. For the input image, the feature maps of 8 times, 16 times, and 32 times down sampling were used to detect targets of different sizes. In the network model, the resolution of the low-level feature map is higher, the target features are obvious, and the target position is more accurate. After multiple convolution operations, the high-level feature map obtains rich semantic information, but it also reduces the resolution of the feature map. Due to the uneven grain size in the images obtained in the actual environment, the three-layer detection layer of the original structure of YOLOv5s has a large down-sampling multiple, which is easy to lose the feature information of small targets, and the high-level feature map is not easy to obtain the feature information of small targets. In this study, by adding a micro-scale feature detection layer, the low-level feature map and the high-level feature map are fused by splicing to detect, which can effectively improve the detection accuracy.

#### 2.3.3. Millet Ear Detection Model Based on Lightweight YoloV5

**Figure 7:**
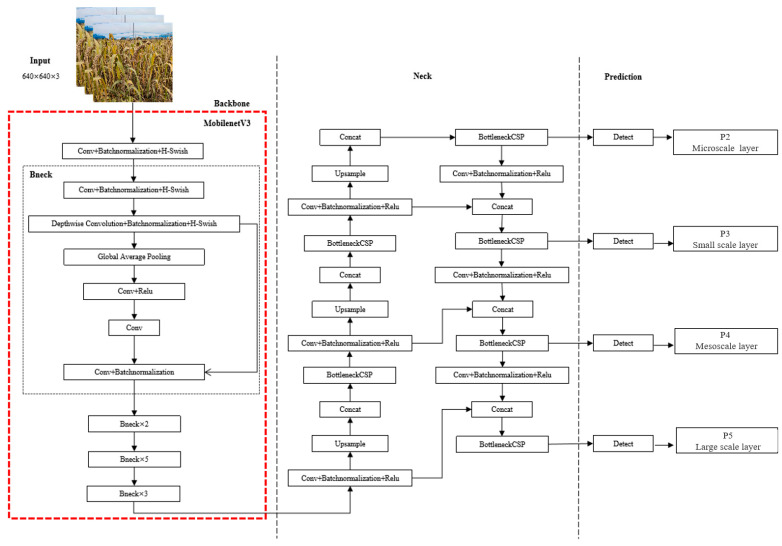
Structure diagram of millet ear detection model based on lightweight YoloV5.

As shown in Figure 7, the structure of the ear detection model based on lightweight YOLOv5 is shown. The adaptive image scaling function of the input end processes the input image into a uniform size of 640 ×640 × 3 and replaces the backbone module of YoloV5 with Mobilenetv3 as a feature extraction network, which can reduce the complexity of the model and reduce the amount of model calculation, but it is also easy to miss overlap and smaller ears. In the multi-feature fusion detection structure, the micro-scale feature detection layer is added to reduce the loss of information during feature fusion, which can better adapt to the detection of millet ears in the complex environment of natural fields, obtain more target information, and improve the detection of small targets. In the post-processing stage, the Merge-NMS algorithm is used to merge the anchor frames by using the position confidence obtained in the feature fusion structure, so as to reduce the false detection and missed detection caused by boundary blurring.

### 2.4. Jetson Nano Platform Test

The experiment was conducted in the laboratory of Shanxi Agricultural University from January 2022 to March 2023. This study was based on the Pytorch deep learning framework for training and testing. The hardware configuration was AMD Ryzen 7 5800 H processor, 6 GB NVIDIA GeForce RTX 3060 Latop GPU. The operating system was Windows 10, 64-bit, Python 3.8.5, CUDA 11.4, cuDNN 8.2.4. The number of batch samples of the model was 4, and the epoch was set to 500. The momentum factor was 0.937, the attenuation coefficient was 0.0005, and the starting learning rate was 0.01.

#### 2.4.1. Evaluating Indicator

In this study, the average detection accuracy (*AP*, %), *F*_1_ score (*F*_1_, %), detection time (s), model size, and floating-point operations (GFLOPs) were used as evaluation indicators. The average detection accuracy is the precision–recall curve (P–R curve), i.e., the area enclosed by the coordinate axis below the curve. The *F*_1_ score is an indicator for comprehensive evaluation of precision and recall rates, reflecting the overall performance of the model. The detection time is the average time for the model to detect an image. The size of the model is the memory space occupied by the model in the system. Floating-point arithmetic is used to reflecte the complexity of the model. The calculation formulas of precision rate (*P*, %), recall rate (*R*, %), *AP* value (%), and *F*_1_ (%) are as follows:

(6) $$P=\frac{TP}{TP+FP}\times 100\%$$

(7) $$R=\frac{TP}{TP+FN}\times 100\%$$

(8) $$AP=\int_{0}^{1}P\left(R\right)dR$$

(9) $$F_{1}=\frac{2\times P\times R}{P+R}\times 100\%$$

where *TP* is a true positive sample, indicating the number of correctly identified millets, *FP* is a false positive sample, i.e., the number of other errors identified as millets, and *FN* is a false negative sample, i.e., the number of unrecognized millet targets.

Because some millet ears were not detected or overlapping millet ears were identified as single during image testing, and there were multiple objects in the images, we calculated the number of detected millet ears as a percentage of the total, which was another parameter used to evaluate the model.

#### 2.4.2. Platform Deployment

**Figure 8:**
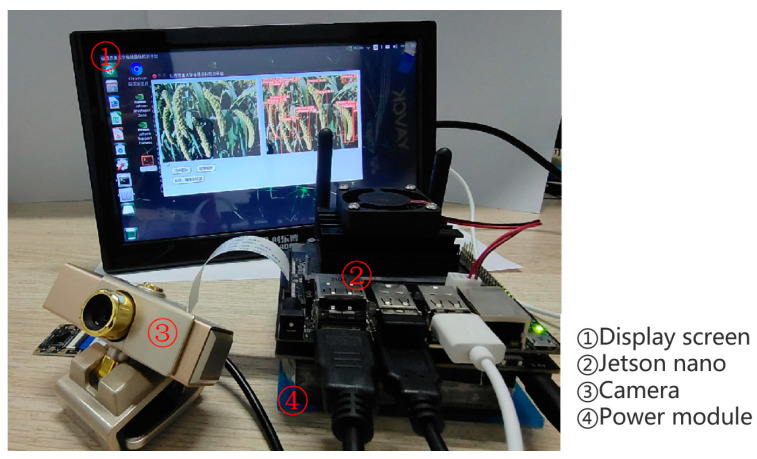
Schematic diagram of Jetson Nano.

YOLOv5s and lightweight improvement model YOLOv5s-MobileNetV3s-multi-scale detection layer-Merge NMS, YOLOv5s-GhostNet-CA-EIOU, and YOLOv5s-ShuffleNetV2-BiFPN-CBAM are deployed in the Jetson Nano for comparison as shown in Figure 8.

**Figure 9:**
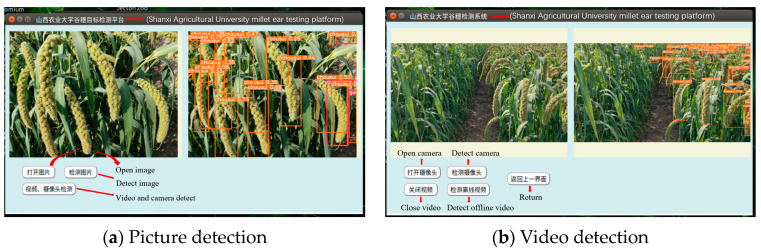
The grain detection system based on the Jetson Nano.

Figure 9 shows the schematic diagram of the image detection and video detection of the grain detection system based on the Jetson Nano development board. The visualization results show that the lightweight model has a good detection effect on the image and video detection in the Jetson Nano detection platform.

## 3. Results and Discussion

### 3.1. Analysis of Training Results

**Figure 10:**
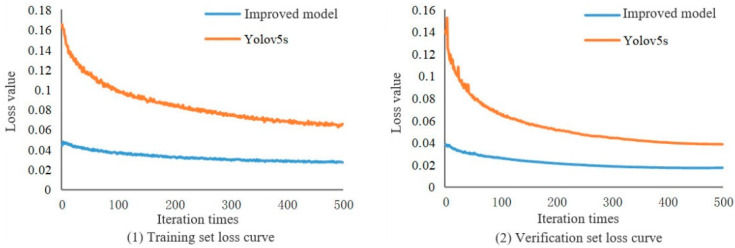
Model training loss value curve.

The change trend of the loss value with the number of iterations reflects the training effect of the model; the closer the loss value is to the end of 0 training, the stronger the model effect. The training loss value curve of the improved YOLOv5s model and the standard YOLOv5s model for this study is shown in Figure 10. It can be seen from the curve in the figure that the loss values of the two models decrease with the increase of the number of training iterations, and gradually tend to be stable. After 200 iterations of the improved model, the training set loss value and the verification set loss value gradually converge, the training set loss value is less than 0.04, the verification set loss value is less than 0.02, and the loss value changes basically smoothly after 300 iterations. The standard model YOLOv5s gradually converges after 350 iterations of the training set loss value and the verification set loss value. After the standard model YOLOv5s tends to be stable, the loss value of the validation set is 59.27% higher than that of the improved model, and the loss value of the validation set is 55.72% higher than that of the improved model. The loss values of the improved model training set and the verification set in this study are closer to 0, indicating that the model training effect is better, and the generalization ability of the whole model is stronger.

### 3.2. Performance Comparison of Model Improvement

**Figure 11:**
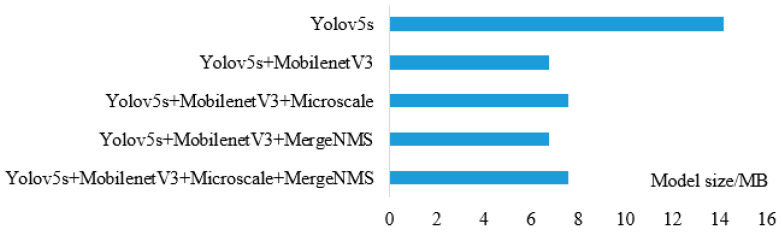
Comparison of model size.

**Figure 12:**
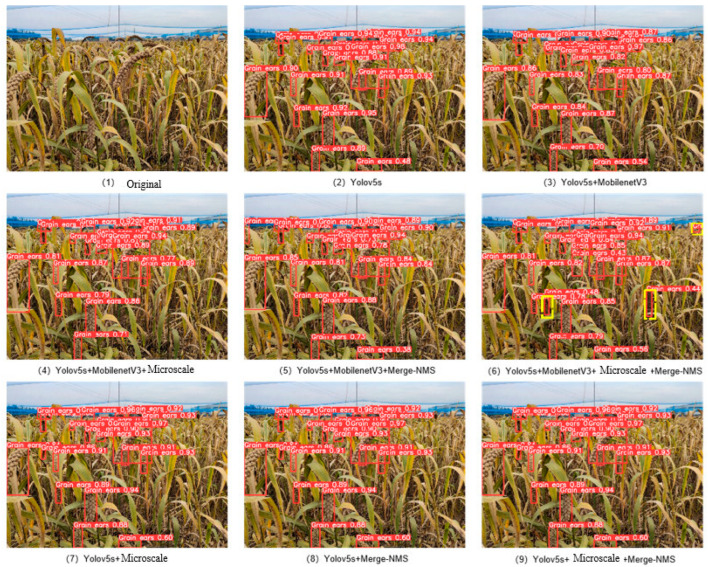
Visual comparison of detection effects of different models.

In order to verify the influence of each improved method on the performance of the model, this study conducts comparative experiments based on the standard YOLOv5s model. The experimental results are shown in Figure 11 and Table 1. Figure 12 shows the visual comparison of the detection effects of different models to reflect the effectiveness of each method on the model.

In this study, MobilenetV3 was used to replace the standard YOLOv5s model backbone structure to reduce the model volume. The experimental results are shown in Figure 11.

The model size of YOLOv5s is 14.19 MB, and the model size of YOLOv5s-MobilenetV3 is 6.77 MB, which is reduced by 7.42 MB. The YOLOv5s-MobilenetV3 model adds micro-scale detection alone to make the detection part of the structure complex, which will slightly increase the size of the model. Compared with YOLOv5s-MobilenetV3, it only increased by 0.79 MB, but still 46.7% smaller than the YOLOv5s model. The Merge-NMS algorithm does not increase the model volume, so the YOLOv5s-MobilenetV3 model volume using the Merge-NMS algorithm alone is 6.77 MB. The improved model in this study is a model composed of two methods on YOLOv5s-MobilenetV3. The model size is 7.56 MB, which is still greatly reduced by 6.63 MB compared with the model size of the standard YOLOv5s model. This proves the effectiveness of MobilenetV3 replacing the backbone structure of YOLOv5s.

From Figure 11 and Table 1, it can be seen that the volume of the YOLOv5s-MobilenetV3 model is greatly reduced compared with that of the YOLOv5s model, and the average detection accuracy is also greatly reduced by 4.2%.

The floating-point operation of the YOLOv5s-MobilenetV3 model is 49.4% less than that of the YOLOv5s model, and the detection time is 0.010 s. It is further proved that replacing the standard YOLOv5s model backbone structure with MobilenetV3 can reduce the model complexity and reduce the detection time. The F1 score of the YOLOv5s-MobilenetV3 model is 5.84% lower than that of the YOLOv5s model, reflecting that the performance of the model structure will also be degraded after lightweight replacement. The micro-scale detection layer is used separately on the YOLOv5s-MobilenetV3 model and the YOLOv5s model. The number of floating-point operations of the YOLOv5s-MobilenetV3 model and the YOLOv5s model has a small increase, indicating that the micro-scale detection layer can increase the complexity of the model to obtain more target information, and the average detection accuracy of the YOLOv5s-MobilenetV3 model is increased from 95.20% to 97.70%, indicating that the micro-scale detection layer can improve the detection accuracy of millet targets in some degree. The percentage of detected millet ears ranged from 78.26% to 91.30% in different models; the YOLOv5s-MobilenetV3-Microscale-MergeNMS combination had the highest value of 91.30%.

In the natural environment, the distribution of grain targets is very dense, and the size targets are alternately distributed, and there are many situations such as grain winding and grain occlusion. The target samples with blurred boundaries may be missed as negative samples, as shown in Figure 12(1).

The evaluation index shows that the *TP* value and *FP* value are directly related to the model performance. In order to improve the detection effect of the model, this study uses the Merge-NMS algorithm to reduce the sample missed detection in the post-processing stage, and the detection results are shown in Figure 12. When the Merge-NMS algorithm is used in the post-processing stage of the YOLOv5s-MobilenetV3 model, the average detection accuracy is increased to 95.56%. The sample data statistics detected in the test set (a total of 2864 samples) are shown in Table 2.

After YOLOv5s-MobilenetV3 adopts the Merge-NMS algorithm, the number of *FN* samples is reduced from 286 to 265. Finally, the number of *FN* samples of the improved model in this study is reduced to 180, and the recall rate is increased from 90.00% to 93.70%, indicating that the Merge-NMS algorithm is effective in solving the problem of target boundary blurring.

After improving the YOLOv5s model with MobilenetV3 lightweight, the complexity of the model is reduced, which makes the feature extraction of the model insufficient. In this study, the micro-scale detection layer is added to the multi-feature fusion detection structure, and the target information extracted from the high-level feature map and the low-level feature map is effectively fused to reduce the loss of target information and improve the detection of small targets. At the same time, the Merge-NMS algorithm can effectively detect targets with fuzzy boundaries in the feature map. As shown in Figure 12(6), the detection visualization effect diagram of the improved model in this study shows that the front ear targets are basically detected and marked, and the occluded ear and the smaller ear in the yellow frame are also successfully detected, indicating that the lightweight model YOLOv5s-MobilenetV3 can effectively improve the detection performance of the model by using both methods.

### 3.3. Comprehensive Comparison of Different Target Detection Networks

**Figure 13:**
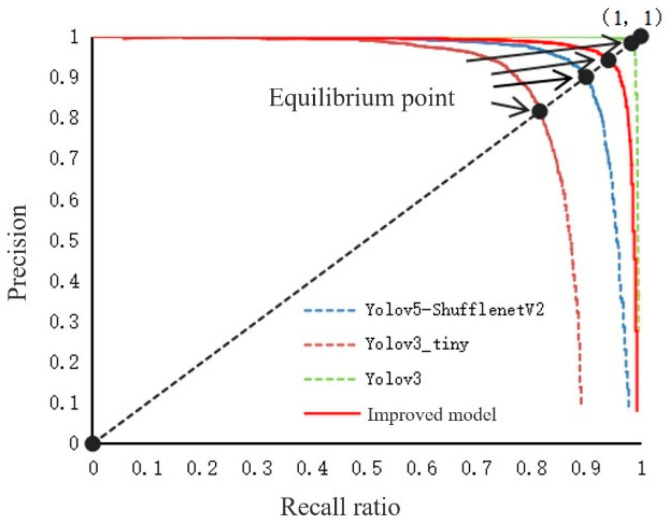
P–R curve of different networks.

In order to verify the effectiveness of the millet detection model in practical applications, classical models such as YOLOv3, YOLOv3-tiny, and YOLOv5-shufflenetV2 were used to compare with the improved model in this study. The experiment uses the same 640 × 640 image as input, sets the same model parameters, and conducts experimental tests on the grain and millet data set self-built in this study. The results are shown in Figure 13 and Table 3.

It can be seen intuitively from Figure 13 that the equilibrium point of the improved model and YOLOv3 model in this study is closer to point (1, 1), and the area under the P–R curve of the improved model and YOLOv3 model in this study is larger than that of other models, i.e., the average detection accuracy is higher. From the comparison of the test results of different models in Table 3, it can be concluded that this study has other advantages while ensuring the accuracy of model detection, such as small model volume and less floating-point operation. The model volume and floating-point operation of the YOLOv5-shufflenetV2 model and the YOLOv3-tiny model are relatively small, but the average detection accuracy is low. The detection accuracy of the YOLOv3 model is high, but the model size reaches 18.05 MB, and the floating-point operation is 2.7 times that of the improved model in this study. The results show that compared with other models, the improved model in this study maintains a balance between detection accuracy and detection speed while reducing model complexity and model volume.

### 3.4. Monitoring Results of Jetson Nano

The test results of improved models on the Jetson Nano development board are shown in Table 4.

The mean average precision of the lightweight model YOLOv5s-MobileNetV3s- Multiscale-MergeNMS was 91.80%, which was slightly lower than the standard YOLOv5s model.The detection speed was 6.95 FPS, indicating that the model maintains a good detection speed after lightweight. The size of the improved model was reduced by 6.63 MB. The comparison of the test results shows that the lightweight improved models on the Jetson Nano can meet the real-time and accuracy requirements of the millet detection applied to the actual environment.

## 4. Conclusions

In this study, a millet detection model based on lightweight YOLOv5s model was proposed. The backbone feature extraction network of YOLOv5s was replaced with a lightweight model MobilenetV3 with attention mechanism. The constructed YOLOv5s-MobilenetV3 model had lightweight characteristics and improves the portability of the model. The micro-scale detection layer was added to the multi-feature fusion detection structure. In the post-processing stage, the Merge-NMS algorithm was used to improve the model to detect various complex scenarios such as dense natural environment, occlusion, and uneven target size distribution. The results showed that the average detection accuracy of the improved model in this study is 97.70%, the *F*_1_ score is 94.20%, and the model size and floating-point operation number are 7.56 MB and 8.5 GFLOPs, which were 46.72% and 49.40% less than the model size and floating-point operation number of YOLOv5s. The average detection time of each image was 0.023 s, which improves the favorable conditions for deployment on embedded mobile platforms, saving human resources and improving work efficiency. The millet ear data set was established according to the natural conditions of the actual environment, and the classical target detection models of YOLOv3, YOLOv3-tiny, and YOLOv5-shufflenetV2 were used for testing and comparison. The modified model in this work maintained good detection performance and ensured the possibility of real-time detection while keeping the model lightweight. The comparison of the test results showed that the lightweight improved models on the Jetson Nano can meet the real-time and accuracy requirements of the millet detection applied to the actual environment. It has a significant impact on the detection of millet growth status and intelligent harvest. Based on the research results of the detection model of millet ears and the deployment of Jetson Nano, we will carry out dynamic estimation of millet yield and improvement and upgrading of intelligent millet harvesting equipment in the future work.

## Figures and Tables

**Table 1:** The influence of improved methods on model performance.

YOLOv5s	Mobilenetv3	Microscale	Merge-NMS	Average Accuracy/%	Precision/%	Recall Ratio/%	*F*_1_/%	Floating-Point	Time/s	Percentage
√				99.40	98.90	98.30	98.60	16.8	0.020	82.61
√	√			95.20	95.70	90.00	92.76	5.9	0.010	86.96
√	√	√		97.70	94.30	93.80	94.05	8.5	0.028	78.26
√	√		√	95.56	95.10	90.70	92.70	5.9	0.015	86.96
√	√	√	√	97.78	94.70	93.70	94.20	8.5	0.023	91.30
√		√		99.40	99.10	97.60	98.34	19.3	0.030	86.96
√			√	99.40	98.80	97.90	98.35	16.8	0.022	86.96
√		√	√	99.40	98.70	97.90	98.30	19.3	0.030	86.96

**Table 2:** Model set test sample statistics.

Model	Precision/%	Recall Ratio/%	*TP*	*FP*	*FN*
YOLOv5s + Mobilenetv3	95.70	90.00	2587	115	286
YOLOv5s + Mobilenetv3 + Merge-NMS	95.10	90.70	2599	135	265
Improved model	94.70	93.70	2684	149	180

**Table 3:** Test results of different models.

Model	Precision/%	Recall Ratio/%	*F*_1_/%	Average Accuracy/%	Size/MB	Floating-Point	Time/s
YOLOv3	98.40	98.00	98.20	99.40	18.05	23.2	0.034
YOLOv3-tiny	90.00	77.60	83.34	84.90	4.22	3.3	0.009
YOLOv5-shufflenetv2	92.40	88.60	90.46	94.20	2.68	3.8	0.012
Improved model	94.70	93.70	94.20	97.70	7.56	8.5	0.023

**Table 4:** Comparison of test results of improved models on the Jetson Nano.

Model	Size/MB	mAP/%	FPS
YOLOv5s	14.19	96.40	6.97
YOLOv5s-MobileNetV3s- Multiscale-MergeNMS	7.56	91.80	6.95

## Data Availability

Data are contained within the article.
